# Antimicrobial susceptibility among gram-positive and gram-negative blood-borne pathogens collected between 2012-2016 as part of the Tigecycline Evaluation and Surveillance Trial

**DOI:** 10.1186/s13756-018-0441-y

**Published:** 2018-12-13

**Authors:** Zhijie Zhang, Meng Chen, Ying Yu, Sisi Pan, Yong Liu

**Affiliations:** 1 Shengjing Hospital of China Medical University, Shenyang, China; 2 Affiliated Hospital of Hebei University, Baoding, China; 3 Pfizer Investment Co.,Ltd, Shanghai, China

**Keywords:** Antimicrobial drug resistance, Blood, Surveillance, tigecycline, Gram-positive bacteria, Gram-negative bacteria

## Abstract

**Background:**

Antimicrobial activity of tigecycline and comparator agents was assessed*in vitro*against 27857 isolates source from blood samples collected between 2012 and 2016 as part of the Tigecycline Evaluation and Surveillance Trial (TEST).

**Methods:**

The broth microdilution methods was used to determine  minimum inhibitory concentrations (MIC) of blood-borne isolates according to guildlines of the Clinical and Laboratory Standards Institute (CLSI). Antimicrobial susceptibility breakpoints from CLSI guidelines were used as standards to determine susceptibility against comparator agents, whereas tigecycline breakpoints were provided by the US Food and Drug Administration (FDA).

**Results:**

More than 91% Enterobacteriaceae isolates, belonging to *Escherichia coli*, *Klebsiella pneumoniae*, *Enterobacter cloacae*and*Serratia marcescens*, were susceptible to amikacin, meropenem, and tigecycline. Meropenem resistance was observed in 8% of*K.pneumoniae* isolates worldwide. Extended-spectrum β-lactamase (ESBL) was produced in 15.9 and 20.9%*E.coli* and *K.pneumoniae*isolates, respectively. MIC_90_ of tigecycline against *Acinetobacter baumannii* was 2 μg/ml.  The highest proportion of susceptible *A.baumannii*isolates was 70.8% for minocycline. Among *P.aeruginose*  isolates worldwide, 71.1–94.9% were susceptible to six antibiotics. Almost all *Staphylococcus aureus*isolates were susceptible to linezolid(100%), vancomycin(100%), and tigecycline (99.9%). The proportion of methicillin-resistant *S.aureus *(MRSA) was 33.0% among *S.aureus*isolates worldwide; it was highest in Asia with 46.6%, followed by North America and Latin America with 37.7 and 34.2%, respectively. Vancomycin-resistant (VR) isolates represented 1.4% of*Enterococcus faecalis* (VR.*E.faecalis*) and 27.6% of *Enterococcus faecium*(VR.*E.faecium*). Highest percentages of VR.*E.faecium*were found in North America and Latin America, with 61.6 and 58.1% of the isolates, respectively. Production of penicillin-resistant *Streptococcus pneumoniae*(PRSP) represented 9.0% of *S. pneumoniae* isolates worldwide; the PRSP proportion was 25.8% in Asia, 13.0% in Africa, and 11.8% in Latin America.

**Conclusions:**

In our study, tigecycline was the only antibiotic that was active against over 90% of all major blood-borne pathogens. A global comparison revealed that antimicrobial resistance was higher in Africa, Asia and Latin America than in Europe and North America.

## Background

Bloodstream infections, acquired in clinics are major cause of mortality in severe disease patients. More attention has been recently paied to bloodstream infections because of the severe effects on health, longer hospital stays, expensive hospitalization costs, and an increase in mortality. In 2013, the incidence of severe sepsis in the United States was approximately 300 cases per 100,000 people associated with a mortality of 20–30% and an expenditure of about $14 billion/year [1].

Importantly, the mortality of bacteremia is directly correlated to the first adequate anti-infectious therapy. In a study on patients of intensive care units (ICU) in Japan,the initiation of an appropriate empirical antimicrobial treatment was associated with a lower 60-day mortality than that of an inappropriate therapy [2]. The selection of an empirical antimicrobial drug therapy should be based on clinical and epidemiological data. Hence, it is important for the clinical treatment of infections to have the information derived from epidemiological data, which differ in scope and focus, i.e., data collected from around the world, different regions,countries, provinces, and hospitals.

The Tigecycline Evaluation and Surveillance Trial (T.E.S.T.), which was initiated in 2004, is a global surveillance study focused on monitoring antimicrobial resistance worldwide. In this study, we report on the antimicrobial susceptibility of Gram-positive bacteria and Gram-negative bacteria isolated from blood specimen collected from around the world between 2012 and 2016.

## Methods

### Isolate collection

Each participating center was required to contribute at least 135 Gram-negative and 65 Gram-positive isolates per study year (Species and number of isolates: *Klebsiella* spp.*,*25; *Escherichia coli*, 25;*Enterobacter* spp., 25; *Pseudomonas aeruginosa*, 20; *Acinetobacter* spp., 15;*Haemophilus influenzae*, 15; and *Serratia* spp., 10; *Staphylococcus aureus*, 25; *Enterococcus* spp.,15;*Streptococcus pneumoniae,*15; and *Streptococcus agalactiae,*10). Isolates were collected from patients with an infectious disease and identified as the causative agent according to laboratory criteria. One isolate per patient was accepted. All body sites were acceptable for sample collection but the use of urine was limited to not more than 25% of all samples. Stored or duplicate isolates were not acceptable.

### Antimicrobial susceptibility testing

Participating centers determined the minimum inhibitory concentrations(MICs) with the broth microdilution assay [3] using MicroScan® panels (Dade MicroScan Inc.,West Sacramento,CA,USA) according to the manufacturer’s instructions. To determine the susceptibility to antimicrobial agents, breakpoints from the Clinical and Laboratory Standards Institute (CLSI) guidelines [4] were used as interpretative standards except for the tigecycline breakpoints, which were obtained from the US Food and Drug Administration(FDA) [5]. The penicillin oral breakpoints (susceptible, ≤0.06 mg/L; resistant,≥2 mg/L) were used for *S. pneumoniae.*Breakpoint were not available for tigecycline against *Acinetobacter baumannii*. Methicillin-resistant *S.aureus*(MRSA) and extended-spectrum β-lactamase(ESBL)-producing *E.coli* and *Klebsiella spp*. were identified by the centers according to the CLSI guidelines [4].

The antimicrobials used in this study were listed in Tables 3 and 4. There were an additional four antimicrobials (azithromycin, clarithromycin, erythromycin and clindamycin) included in the panel for testing *S.pneumoniae*.

### Quality control

The reference laboratory, International Health Management Associates(IHMA, Schaumburg, IL, USA), was responsible for the coordination of isolate collection, transport, and backup, as well as the administration of a database. Approximately 10–15% of the isolates were randomly selected each year by IHMA to verify isolate identity and MICs.

## Results

### Isolate collection

Between 2012 and 2016, 27,857 isolates, including 17,237 Gram-negative (61.9%) and 10,620(38.1%) Gram-positive isolates, were recovered from globally collected blood samples. The major Gram-negative bacteria were *E. Coli*(*n* = 5352;19.2%), *K.pneumoniae *(*n* = 3154;11.3%),*E. cloacae*(*n* = 1824;6.6%), *P .aeruginosa*(*n* = 1739;6.2%), *S.marcescens *(*n* = 1024;3.7%) and *A.baumannii *(n=749;2.7%). *S.aureus *(*n* = 3324;11.9%), *S.pneumoniae *(*n* = 1983;7.1%), *E.feacalis *(*n* = 1527;5.5%), *E.faecium *(*n* = 1000;3.6%) and *S.agalactiae *(*n* = 982;3.5%), were the dominating Gram-positive bacteria.

Europe and North America were the regions with the most participating centers and, therefore,contributed more isolates than the other regions. There were 132 and 54 participating centers collecting 17,456(62.7%) and 6785(24.3%) isolates in Europe and North America,respectively (Table 1). From all patients, 71.5% of the subjects were from non-ICUs and, 70.1% received in-patient treatment. The proportion of subjects of 61-80 years of age was 49.1% (Table 2).

**Table 1:** Participating centers and collected isolate per world region

Region^a^	Number of Centers	Percent of centers (%)	Number of isolates	Percent of isolates (%)
Africa	8	3.4	375	1.4
Asia	29	12.1	1989	7.1
Europe	132	55.2	17,456	62.7
North America	54	22.6	6785	24.3
LatinAmerica	16	6.7	1252	4.5
Total	239	100	27,857	100

^a^Africa:= Egypt, Morocco, South Africa, Tunisia; Asia: =China, Kuwait, Hong Kong, India, Japan, Jordan, Saudi Arabia, South Korea, Malaysia, Pakistan, Philippines, Singapore, Taiwan, Thailand, Vietnam; Europe:=Austria, Belgium, Croatia, Czech Republic, Denmark, Finland, France, Germany, Greece, Hungary, Ireland, Italy, Lithuania, Latvia, Netherlands, Poland, Portugal, Romania, Serbia, Spain, Sweden, Switzerland, United Kingdom; Latin America: = Argentina, Brazil, Chile, Colombia, Mexico, Panama, Guatemala,Venezuela; North American: =Canada, United States

**Table 2:** Distribution of patients according to location and age

Demographic parameter	Number of patients	Percent of patients(%)
Patient location		
ICU^a^	5419	19.5
non-ICU	19,907	71.5
Unknown	2531	9.1
In-patient	19,520	70.1
Out-patient	5806	20.8
Unknown	2531	9.1
Patient age		
0–20 years	2627	9.4
21–40 years	2471	8.9
41–60 years	6507	23.4
61–80 years	10,901	39.1
≥ 81 years	4674	16.8
Unknown	677	2.4
Total	27,857	100

^a^*ICU*= intensive care unit

### Antimicrobial susceptibilities of isolates

#### Enterobacteriaceae

**Fig. 1:**
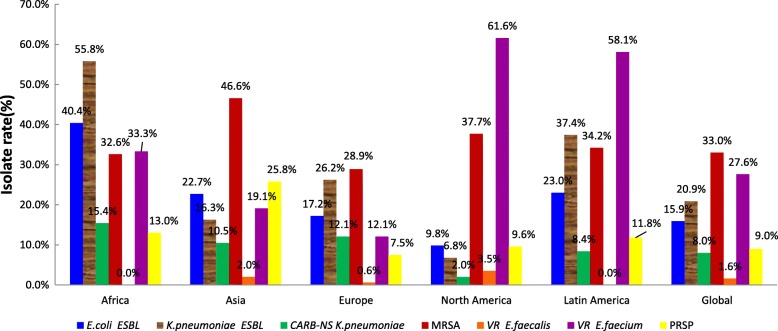
Distribution of multi-drug resistant (MDR) bacteria among isolates from blood specimen collected in various international regions. Percent on every column indicates percentage of resistant isolates in each region for each organism. ESBL, extended-spectrum β-lactamase; CARB-NS, cabapanem non-susceptibility; MRSA, methicillin-resistant *S. aureus*; VR, vancomycin resistant; PRSP, Penicillin-resistant *S. pneumoniae*

Four species of Enterobacteriaceae were isolated from bloodspecimens, *E.coli*, *K.pneumoniae*, *E.cloacae,* and*S.marcescens*; most of these isolates (99,91,95, and 96%, respectively)were susceptible to amikacin, meropenem, and tigecycline. The most meropenem-resistant isolates were found in*K.pneumoniae,* with 8% of resistant isolates globally. A lower proportion of resistant isolates was found in *E.cloacae, S.marcescens,* and *E.coli,* with only 1.6,1.6, and 0.4% of the isolates, respectively. There were large differences in the occurrence of meropenem-resistant*K.pneumoniae*, which was highest in Africa (15.4%), followed by Europe, Asia, and Latin America (12.1,10.5, and 8.4%, respectively), and lowest in North America (2%) (Table 3, Fig. 1).

**Table 3:** Antimicrobial susceptibility among Gram negative isolates including ESBL-producing strains from blood specimens collected between 2012 and 2016

	Africa	Asia	Europe	North America	Latin America	Global				Global			
	S%	S%	S%	S%	S%	MIC_90_(mg/L)	MIC range(mg/L)	S%	R%	MIC_90_(mg/L)	MIC range(mg/L)	S%	R%
	*E.coli*					*E.coli,* all isolates				*E.coli* ESBL isolates			
	*N* = 47	*N* = 396	*N* = 3235	*N* = 1487	*N* = 187	N = 5352				*N* = 852			
Amikacin	97.9	97.7	99.5	99.9	98.9	4	0.5–128	99.4	0.2	8	0.5–64	98.4	0.2
Amoxy/clav	48.9	72.0	70.1	78.1	64.7	32	0.25–64	72.1	10.1	32	0.5–64	48.6	14.4
Ampicillin	12.8	18.4	32.8	43.4	26.2	64	0.5–64	34.3	65.0	64	1–64	0.6	99.4
Cefepime	57.5	59.6	82.0	88.8	73.8	16	0.5–64	81.8	13.1	64	0.5–64	13.9	64.7
Ceftazidime	63.8	70.0	86.5	91.6	79.1	8	1–32	86.3	9.4	32	1–32	35.3	42.7
Ceftriaxone	48.9	49.8	78.8	87.4	67.4	64	0.06–64	78.4	21.0	64	0.06–64	0.9	97.8
Levofloxacin	51.1	48.0	64.9	71.6	58.8	16	0.008–16	65.2	31.9	16	0.008–16	16.3	78.9
Meropenem	95.7	98.2	99.9	99.7	97.3	0.06	0.06–32	99.6	0.4	0.12	0.06–16	99.5	0.4
Minocycline	74.5	83.3	85.9	91.3	81.8	8	0.5–32	87.0	7.4	8	0.5–32	86.0	7.3
Pip/taz	80.9	95.0	92.7	97.4	94.4	8	0.06–256	94.1	3.2	16	0.06–256	91.2	4.5
Tigecycline	100	100	99.9	99.9	100	0.25	0.008–16	99.9	0.04	0.5	0.008–2	100	0.0
	*K.pneumoniae*					*K.pneumoniae,* all isolates				*K.pneumoniae* ESBL isolates			
	*N* = 52	*N* = 276	*N* = 1761	*N* = 899	*N* = 166	N = 3154				*N* = 659			
Amikacin	84.6	90.2	96.7	99.4	96.4	8	0.5–128	96.7	1.8	16	0.5–128	95.8	2.7
Amoxy/clav	25.0	66.7	60.8	90.9	59.0	64	0.12–64	69.2	18.8	64	2–64	19.6	40.2
Ampicillin	1.8	2.2	2.6	6.2	1.2	64	0.5–64	3.5	85.1	64	16–64	0	99.9
Cefepime	34.6	66.7	65.0	93.3	57.2	64	0.5–64	72.3	23.4	64	0.5–64	8.4	78.0
Ceftazidime	34.6	69.2	67.0	91.2	68.7	32	1–32	73.6	21.7	32	1–32	18.1	65.0
Ceftriaxone	21.2	62.7	62.5	90.6	54.2	64	0.06–64	69.4	29.8	64	0.06–64	1.8	97.3
Levofloxacin	57.7	76.5	70.8	91.4	80.7	16	0.008–16	77.5	18.7	16	0.03–16	40.5	47.7
Meropenem	84.6	89.5	87.9	98.0	91.6	1	0.06–32	91.1	8.0	8	0.06–32	84.8	11.7
Minocycline	71.2	83.3	78.6	87.5	83.7	16	0.5–32	81.7	11.5	16	0.5–32	70.3	18.2
Pip/taz	55.8	80.8	76.7	94.1	77.1	256	0.06–256	81.7	15.0	256	0.5–256	59.6	29.1
Tigecycline	96.2	93.1	94.4	95.6	97.6	2	0.008–16	94.8	0.7	2	0.008–16	90.4	1.2
	*S.marcescens*												
	*N* = 29	*N* = 88	*N* = 591	*N* = 224	*N* = 92	N = 1024							
Amikacin	100.0	96.6	99.0	100.0	85.9	4	0.5–128	97.9	0.9				
Amoxy/clav	0.0	8.0	3.1	1.3	3.3	64	0.5–64	3.0	92.4				
Ampicillin	0.0	6.8	6.3	4.0	4.4	64	0.5–64	5.5	87.3				
Cefepime	89.7	85.2	96.6	98.7	80.4	1	0.5–64	94.4	3.8				
Ceftazidime	96.6	88.6	96.1	98.2	87.0	2	1–32	95.1	3.8				
Ceftriaxone	89.7	79.6	85.3	88.0	71.7	4	0.06–64	84.3	11.8				
Levofloxacin	100.0	94.3	95.8	96.0	89.1	1	0.008–16	95.2	2.9				
Meropenem	96.6	95.5	99.0	99.1	93.5	0.25	0.06–32	98.1	1.6				
Minocycline	100.0	88.6	90.4	94.2	88.0	4	0.5–32	91.9	2.3				
Pip/taz	100.0	89.8	94.1	97.3	90.2	8	0.06–256	94.2	2.4				
Tigecycline	96.6	95.5	97.0	97.8	93.5	2	0.03–16	96.7	0.3				
	*E.cloacae*												
	N = 47	*N* = 157	*N* = 1084	*N* = 404	*N* = 132	N = 1824							
Amikacin	95.7	96.8	99.0	100.0	97.0	4	0.5–128	98.8	0.9				
Amoxy/clav	2.1	1.9	3.7	3.0	1.5	64	0.12–64	3.2	95.4				
Ampicillin	4.3	3.2	6.8	4.5	4.6	64	0.5–64	5.8	89.5				
Cefepime	51.1	76.4	77.2	86.9	74.2	16	0.5–64	78.4	12.0				
Ceftazidime	51.1	63.1	65.0	74.5	65.9	32	1–32	66.7	29.6				
Ceftriaxone	40.4	59.2	59.7	70.5	60.6	64	0.06–64	61.6	36.2				
Levofloxacin	91.5	87.9	85.8	94.6	86.4	4	0.008–16	88.1	9.8				
Meropenem	100.0	94.3	97.2	99.5	97.7	0.25	0.06–32	97.6	1.6				
Minocycline	85.1	84.1	86.0	86.9	88.6	8	0.5–32	86.2	8.2				
Pip/taz	78.7	75.2	76.3	81.7	79.6	128	0.06–256	77.7	11.5				
Tigecycline	95.7	96.8	95.4	95.5	97.0	2	0.008–16	95.7	0.8				
	*P.aeruginosa*												
	N = 29	*N* = 150	*N* = 1081	*N* = 387	*N* = 95	N = 1739							
Amikacin	96.6	95.3	94.3	97.9	88.4	8	0.5–128	94.9	2.7				
Cefepime	65.5	76.0	75.5	86.3	85.3	16	0.5–64	78.4	9.1				
Ceftazidime	79.3	77.3	78.8	88.9	82.1	32	1–32	81.1	11.4				
Levofloxacin	86.2	82.7	72.4	76.5	75.8	16	0.015–16	74.6	19.8				
Meropenem	75.9	69.3	69.6	75.7	69.5	16	0.06–32	71.1	22.0				
Pip/taz	82.8	78.0	78.3	86.8	85.3	64	0.12–256	80.7	7.9				
	*A.baumannii*												
	N = 32	*N* = 123	*N* = 424	*N* = 118	N = 52	*N* = 749							
Amikacin	31.3	43.1	37.0	74.6	28.9	128	0.5–128	43.1	49.1				
Cefepime	12.5	27.6	24.1	54.2	15.4	64	0.5–64	28.3	65.7				
Ceftazidime	15.6	35.0	27.1	50.0	23.1	32	1–32	31.2	64.2				
Ceftriaxone	9.4	22.8	16.0	31.4	11.5	64	0.06–64	19.0	67.0				
Levofloxacin	21.9	30.9	23.6	51.7	15.4	16	0.008–16	28.6	61.7				
Meropenem	25.0	26.8	26.9	60.2	15.4	32	0.06–32	31.2	66.8				
Minocycline	81.3	73.2	61.8	89.0	90.4	8	0.5–32	70.8	8.8				
Pip/taz	12.5	26.0	24.5	52.5	13.5	256	0.06–256	27.9	68.2				
Tigecycline						2	0.008–4						

Abbreviations: *S%* percentage of susceptible isolates, *R%* percentage of resistant isolates, *MIC* minimum inhibitory concentration, *Amoxy/clav* Amoxicillin/clavulanic acid, *Pip/taz* Piperacillin/tazobactam

Susceptibility to cephalosporins(ceftriaxone,ceftazidime, and cefepime) was found in 78.4–86.3%, 69.4–73.6%, 61.6–78.4%, and 84.3–95.1% of isolates of*E.coli, K.pneumoniae, E.cloacae, * and *S.marcescens*, respectively. The most cephalosporin-susceptible organism was *S.marcescens,*whereas *K.pneumoniae* and *E.cloacae*were less susceptible. In the four species of Enterobacteriaceae, *E.coli, K.pneumoniae, E.cloacae,* and *S.marcescens*, susceptibility to piperacillin/tazobactam(pip/taz) and amoxicillin/clavulanic acid(amoxy/clav) was found in 94.1%/72.1, 81.7%/69.2, 77.2%/3.2, and 94.2%/3.0% of the isolates, respectively. The in vitro activity of pip/taz was lower against *K. pneumoniae* and *E. cloacae* than *E. coli* and *S .marcescens*. The activity of amoxy/clav against *E. coli* and *K. pneumoniae* was weak as compared to that of pip/taz*,* and it had almost no effect on *E. cloacae* and *S. marcescens* (Table 3)*.*

Minocycline was more active against the four Enterobacteriaceae with a similar susceptibility in 87.0, 81.7, 86.2, and 91.7% of the isolates of *E. coli, K .pneumoniae, E .cloacae,*and *S. marcescens,*respectively. The activity of levofloxacin was lowest against *E. coli*(65.2%) and highest against *S. marcescens*(95.2%) (Table 3).

ESBL production was found in 15.9% of *E. coli* and 20.9% of *K. pneumoniae* isolates. Africa had the highest proportion of ESBL-producers, with 40.4%(19/47) and 55.8%(29/52) of *E. coli* and *K. pneumoniae* isolates, respectively, followed by Latin America(23.0%,43/187), Asia(22.7%,90/396), Europe(17.2%,555/3235), and North America(9.8%,145/1487) in *E. coli,*as well as Latin America(37.4%,62/166), Europe (26.2%,462/1761), Asia (16.3%,45/276), and North America(6.8%,61/899) in *K. pneumoniae *(Fig. 1)*.*

Susceptibility to amikacin, meropenem, and tigecycline was found in more than 98.4% of ESBL-producing *E. coli* isolates, as well as in 95.8,84.8 and 90.4% of ESBL-producing *K.pneumoniae* isolates, respectively. Over than 86% of ESBL-producing *E. coli* isolates were susceptible to pip/taz and minocycline*,*but only 59.6–70.3% of ESBL-producing *K. pneumoniae.* Susceptibility to other antibiotics was observed in less than 50% of Enterobacteriaceae isolates (Table 3).

#### *A. baumannii* and *P. aeruginosa*

Antibiotics exhibited a very poor activity against *A. baumannii.* The most active antibiotic was minocycline; 70.8% of *A. baumannii* isolates were susceptible. Susceptibility to meropenem was found in 31.2% of the isolates. The MIC_90_ of tigecycline was 2 μg/ml (Table 3).

Amikacin was the most active antibiotic in *P. aeruginose,* i.e., 94.9% susceptible isolates*.* Susceptibility to ceftazidime and pip/taz was found in over 80% of the isolates. Susceptibility to other antibiotics was confirmed for over 70% of the isolates (Table 3).

#### *S. aureus*

Almost all *S. aureus* isolates were susceptible to linezolid(100%), vancomycin(100%), and tigecycline(99.9%). The isolates were also highly susceptible to minocycline (98.8%). The lowest susceptibility in *S. aureus* was observed for penicillin; only14.7% of susceptible isolates (Table 4). Moreover, the proportion of MRSA was 33.0% globally; it was highest in Asia with 46.6%, followed by North America(37.7%) and Latin America (34.2%), whereas the MRSA percentage was lower in Africa(32.6%) and Europe(28.9%) (Fig. 1).

**Table 4:** Antimicrobial susceptibility among Gram-positive isolates including multi-drug resistance(MDR) strains from blood specimens collected between 2012 and 2016

	Africa	Asia	Europe	North America	Latin America	Global				Global			
	S%	S%	S%	S%	S%	MIC_90_(mg/L)	MIC range(mg/L)	S%	R%	MIC_90_(mg/L)	MIC range(mg/L)	S%	R%
	*S.aureus*				*S.aureus*, all isolates					MRSA			
	*N* = 43	*N* = 298	*N* = 2008	*N* = 814	*N* = 161	N = 3324				*N* = 1096			
Levofloxacin	65.1	57.4	69.3	67.3	77.0	16	0.06–64	68.1	30.2	64	0.06–64	21.9	75.6
Linezolid	100.0	100.0	100.0	100.0	100.0	2	0.5–4	100.0	0.0	2	0.5–4	100.0	0.0
Minocycline	83.7	97.3	99.5	98.3	100.0	0.25	0.25–16	98.8	0.1	0.5	0.25–16	97.5	0.1
Penicillin	2.3	6.0	15.9	16.6	8.1	16	0.06–16	14.7	85.4	16	0.12–16	0.1	99.9
Tigecycline	100	99.7	100.0	100.0	100.0	0.12	0.008–1	99.9	0.0	0.25	0.015–1	99.9	0.0
Vancomycin	100.0	100.0	100.0	100.0	100.0	1	0.12–2	100.0	0.0	1	0.12–2	100.0	0.0
	*E.faecalis*				*E.faecalis*, all isolates					VR *E.faecalis*			
	*N* = 26	*N* = 99	*N* = 910	*N* = 406	*N* = 86	N = 1527				N = 22			
Ampicillin	100.0	96.0	98.9	99.8	96.5	1	0.06–32	98.8	1.2	32	0.5–32	86.4	13.6
Levofloxacin	69.2	70.7	68.6	73.4	81.4	64	0.06–64	70.7	28.5	64	0.5–64	13.6	86.4
Linezolid	100.0	97.0	100.0	100.0	97.7	2	0.5–8	99.7	0.1	2	0.5–2	100.0	0.0
Minocycline	30.8	35.4	36.8	35.2	38.4	8	0.25–16	36.3	6.3	8	0.25–8	27.3	0.0
Penicillin	100.0	95.0	98.8	99.8	96.5	4	0.06–16	98.7	1.3	16	2–16	86.4	13.6
Tigecycline	100.0	100.0	99.9	100.0	100.0	0.12	0.008–0.5	100.0	0.0	0.12	0.03–0.12	100.0	0.0
Vancomycin	96.2	98.0	99.0	96.6	98.8	2	0.12–64	98.2	1.4	64	32–64	0.0	100.0
	*E.faecium*				*E.faecium*, all isolates					VR *E.faecium*			
	N = 9	N = 89	*N* = 603	*N* = 268	N = 31	N = 1000				N = 276			
Ampicillin	11.1	12.4	11.1	14.2	6.5	32	0.06–32	11.9	88.1	32	4–32	0.7	99.3
Levofloxacin	22.2	9.0	9.8	12.3	12.9	64	0.06–64	10.6	87.0	64	8–64	0.0	100.0
Linezolid	100.0	100.0	99.7	98.8	100.0	2	0.5–8	99.5	0.2	2	0.5–8	98.9	0.4
Minocycline	88.9	47.2	70.2	59.0	80.7	8	0.25–16	65.6	6.2	8	0.25–16	58.7	6.2
Penicillin	11.1	14.6	13.1	12.7	6.5	16	0.06–16	12.9	87.1	16	4–16	1.5	98.6
Tigecycline	100.0	100.0	99.5	98.9	100.0	0.12	0.008–1	99.4	0.0	0.12	0.008–1	98.9	0.0
Vancomycin	66.7	78.7	87.1	38.1	41.9	64	0.12–64	71.6	27.6	64	32–64	0.0	100.0
	*S.pneumoniae*				*S.pneumoniae*, all isolates					PR *S.pneumoniae*			
	*N* = 23	*N* = 66	*N* = 1248	*N* = 553	*N* = 93	N = 1983				*N* = 178			
Amoxy/clav	91.3	95.5	98.0	96.2	93.6	1	0.03–16	97.1	1.1	8	0.03–16	68.5	11.8
Azithromycin	69.6	38.1	81.1	63.0	44.6	64	0.03–512	72.7	26.0	128	0.03–512	28.7	69.0
Ceftriaxone	100.0	94.0	98.7	99.0	98.9	0.5	0.03–8	98.6	0.1	2	0.03–8	86.0	1.1
Clarithromycin	69.6	39.7	80.9	63.0	69.6	128	0.015–128	73.9	26.0	128	0.015–128	30.5	69.0
Clindamycin	78.3	61.9	85.3	87.4	79.4	128	0.015–128	84.7	14.8	128	0.015–128	53.5	45.4
Erythromycin	69.6	39.7	80.8	63.6	69.6	64	0.015–128	74.0	25.8	128	0.015–128	29.9	69.0
Levofloxacin	100.0	98.5	99.6	99.6	98.9	1	0.06–16	99.6	0.4	1	0.12–16	99.4	0.6
Linezolid	100.0	100.0	100.0	100.0	100.0	1	0.5–2	100.0	0.0	1	0.5–2	100.0	0.0
Meropenem	81.0	60.6	89.7	87.0	86.0	0.5	0.12–8	87.8	7.1	1	0.12–8	3.4	70.8
Minocycline	73.9	37.9	77.5	83.7	69.9	4	0.25–16	77.5	16.8	8	0.25–16	46.1	45.0
Penicillin	69.6	34.9	70.4	69.8	55.9	1	0.06–16	68.3	9.0	4	2–16	0.0	100.0
Tigecycline	100.0	98.5	99.8	99.5	100.0	0.03	0.008–4	99.7	0.0	0.03	0.008–0.12	100.0	0.0
Vancomycin	100.0	100.0	100.0	100.0	100.0	0.5	0.12–1	100.0	0.0	0.5	0.12–1	100.0	0.0
	*S.agalactiae*												
	N = 8	*N* = 64	*N* = 505	*N* = 373	N = 32	N = 982							
Ampicillin	100.0	100.0	100.0	100.0	100.0	0.12	0.06–0.25	100.0	0.0				
Ceftriaxone	100.0	100.0	100.0	100.0	100.0	0.12	0.03–0.5	100.0	0.0				
Levofloxacin	100.0	95.3	98.2	96.5	87.5	1	0.06–64	97.1	2.7				
Linezolid	100.0	100.0	100.0	100.0	100.0	1	0.5–2	100.0	0.0				
Meropenem	100.0	100.0	100.0	100.0	100.0	0.12	0.12–0.5	100.0	0.0				
Minocycline	12.5	20.3	17.8	22.0	50.0	16	0.25–16	20.6	62.7				
Penicillin	100.0	100.0	100.0	100.0	100.0	0.12	0.06–0.12	100.0	0.0				
Tigecycline	100.0	96.9	99.8	100.0	100.0	0.06	0.008–4	99.7	0.0				
Vancomycin	100.0	100.0	100.0	100.0	100.0	0.5	0.12–1	100.0	0.0				

Abbreviations: *S%* percentage of susceptible isolates, *R%* percentage of resistant isolates, *MIC* minimum inhibitory concentration, *Amoxy/clav* Amoxicillin/clavulanic acid

#### *E. faecalis* and *E. faecium*

In this study*, E. faecalis was* susceptible to five (penicillin, ampicillin, tigecycline, linezolid, and vancomycin) out of seven antibiotics at a rate of ≥98%. The susceptibility of *E. faecium* was 99.4 and 99.5% to tigecycline and linezolid, respectively, but only 12.9 and 11.9% to penicillin and ampicillin, respectively. The susceptibility of *E. faecium* to vancomycin was 71.6, and 70.7% of *E. faecalis* and 10.6% of *E .faecium* isolates were susceptible to levofloxacin. Further, 36.3% of *E. faecalis* and 65.6% of *E.faecium* isolates were susceptible to minocycline (Table 4).

The isolate rates of vancomycin-resistant *E. faecalis*(VR *E. faecalis*) and *E. faecium*(VR *E. faecium*) were 1.4 and 27.6%, respectively. Most VR E. *faecium* isolates were collected in North America and Latin America with isolate rate of 61.6 and 58.1%, respectively (Fig. 1). Tigecycline and linezolid were active against 98.9% of VR E. *faecium*.

#### *S. pneumoniae*

No linezolid- or vancomycin-resistant isolates were found in our study. Over than 97% susceptibility was observed for tigecycline, levofloxacin, ceftriaxone, and amoxy/clav. Susceptibility to clindamycin was 84.7%, which was higher than that to macrolides, including erythromycin,clarithromycin and azithromycin with similar susceptibility(72.7–74.0%). Meropenem and minocycline were active in 87.8 and 77.5% of the isolates, respectively (Table 4).

The lowest susceptibility among all antibiotics was observed for penicillin with 68.3% of susceptible isolates. The global rate for penicillin-resistant *S. pneumoniae* (PRSP) isolates was 9.0% in globally; regionly, it was 25.8% in Asia,13.0% in Africa,11.8% in Latin America, 9.6% in North America and 7.5% in Europe (Fig. 1).The activity of many antibiotics decreased in PRSP. The rate of isolates susceptible to macrolides decreased to 28.7–30.5% and clindamycin, meropenem, and minocycline were reduced to 53.5, 3.4 and 46.1%, respectively. However, 100% susceptibility was observed for linezolid, vancomycin, tigecycline, and levofloxacin (Table 4).

#### *S. agalactiae*

The proportion of antimicrobial-susceptible*S. agalactiae* isolates was higher than 97%, except for minocycline with 20.6% of susceptible isolates*.* A susceptibility of 100% was found for penicillin, ampicillin, linezolid, meropenem, and vancomycin (Table 4).

#### Variations insusceptibility between the major world regions

Antibiotics with a global susceptibility rate of less than 90% were selected and the ones with the lowest susceptibility rate in two regions were marked. The occurrence of each region due to the marked antibiotics reflected the situation of antimicrobial resistance in this region (Table 5). Africa was the region with the most occurrences of marked antibiotics, 38 times and at the proportion of 34.2%, followed by Latin America (27,24.3%) and Asia(26,23.4%). The proportion was less than 12% for Europe and North America.

**Table 5:** Occurrence of every regions with low susceptibility to antibiotics in major blood-borne pathogens (%)

bacterial	number of antibiotics^a^ × 2	Africa	Asia	Europe	North America	Latin America
*E. coli*	14	7 (50.0)	4 (28.6)	0 (0.0)	0 (0.0)	3 (14.3)
*K .pneumoniae*	16	8 (50.0)	0 (0.0)	3 (18.8)	0 (0.0)	5 (31.3)
*S. marcescens*	6	2 (33.1)	1 (16.7)	0 (0.0)	2 (33.3)	1 (16.7)
*E. cloacae*	16	5 (31.3)	6 (37.5)	2 (12.5)	0 (0.0)	3 (18.8)
*P. aeruginosa*	10	1 (10.0)	3 (30.0)	4 (40.0)	0 (0.0)	2 (20.0)
*A. baumannii*	16	7 (43.8)	1 (6.3)	1 (6.3)	0 (0.0)	7 (43.8)
*S. aureus*	4	2 (50.0)	2 (50.0)	0 (0.0)	0 (0.0)	0 (0.0)
*E. faecalis*	4	2 (50.0)	0 (25.0)	1 (25.0)	1 (25.0)	0 (0.0)
*E. faecium*	11^b^	2^b^(18.2)	2 (18.2)	2^b^(18.2)	2 (18.2)	3 (27.3)
*S. pneumoniae*	14	2 (14.3)	7 (50.0)	0 (0.0)	2 (14.3)	3 (21.4)
Total	111	38 (34.2)	26 (23.4)	13 (11.7)	7 (6.3)	27 (24.3)

^a^ There were antibiotics with a global susceptibility of less than 90%

^b^ Same susceptibility in Africa and Europe of ampicillin to *E. faecium*

## Discussion

Bacteria isolated from blood are typically the causative agents for a circulatory system infection or a local infection(e.g., the special respiratory system) [6], and they often indicate poor prognosis [7]. Therefore, bacteria isolated from blood are important for physicians and studies on pathogenic bacteria and their resistance to antibiotics have a great practical significance and a high clinical value.

Bacterial species isolated from blood specimens in our study were common pathogens of community-acquired and hospital-acquired bloodstream infection [8, 9]. The patients were mostly from non-ICU departments and had the highest proportion in elderly patients 61–80 years of age. A surveillance program performed for 18 years by a large hospital of Malawi in Africa pathogens from bloodstream infections [8] found that the organisms causing differed with the age. Except for children below 4 years of age, bacterial infections were likely to be caused by *Salmonella typhimurium*, *Salmonella enteritidis*,*S. pneumoniae,* and yeast in adults of less than 60 years old of age. Morbidity in bloodstream infections caused by Enterobacteriaceae(such as *E. coli* or *K. pneumoniae)* and *S. aureus* increased with the age and occurred most frequently in elderly people ≥60 years old of age. In this investigation,the proportion of Enterobacteriaceae and *S. aureus* was 52.7% and the trend of the occurrence of the infection increased with age; these observations are in agreement with the findings in the study from Africa.

The high activities determined for amikacin, meropenem, and tigecycline against Enterobacteriaceae from blood samples in our study indicated that these antibiotics can be used as the first choice in the empirical treatment of clinical bloodstram infections caused by Enterobacteriaceae.

Low in vitro activity was observed for cephalosporin and pip/taz against *K. pneumonia*. ESBL production was higher in *K. pneumoniae* than in *E. coli* (20.9% vs. 15.9%). Meropenem resistance found globally in 8% of *K. pneumoniae*, far exceeds that in other Enterobacteriaceae. These results indicated that the lower activity of cephalosporins and pip/taz against *K. pneumoniae* is related to the production of ESBL and carbapenemases.

ESBL was the main reason for *E. coli* and *K. pneumoniae* resistance against the third and fourth generation of cephalosporins because of the hydrolytic activity [10]. Production of ESBL monitored by many large disease surveillance organizations. In this study, the production of ESBL in *E. coli* and *K. pneumoniae* were 22.7%/16.3%, 23.0%/37.4% and 9.8%/6.8% in Asia, Latin America, and North America, respectively. The data from the 2011–2014 SENTRY surveillance, ESBL in *E. coli* and *K. pneumoniae* in Asia-Pacific [11], Latin America [12], and the USA [13] were 60%/47%,37.7%/57.3%, and 11.1%/20.4%, respectively, which was higher than the data in our study. In a meta- analysis from West Africa [14], ESBL in *E. coli* and *K. pneumoniae* were11.9 and 24.2%, respectively, which was less than 40.4 and 55.8% in our study. In a report of the TEST study between 2004 and 2013 [15], ESBL of *E. coli* and *K. pneumoniae* were 14.0 and 20.4% globally, similar to 15.9 and 20.9%, respectively, in our study. Different procedures implemented by different organizations involved in various surveillance programs lead to disparities in surveillance data specific for each region. It is critical to organize very active surveillance stations and collect accurate data to assess the true status of antimicrobial resistance in a local area.

Carbapenemases cause carbapenem resistance in Enterobacteriaceae [16]. *K. pneumoniae* carbapenemases(KPC) and metallo-β-lactamases(MBL)are common epidemic carbapenemases. KPC is reportedly prevalent in Latin-America, Europe and the Middle-East and the MBL is also prevalent in Europe and Asia [16]. No article has mentioned an increase of carbapenemases in Africa, but we found the highest rate of non-susceptibility to carbapenem(15.4%) in Africa. The number of participating centers and the collected strains were minimal in Africa(8 centers; 47*E. coli* and 52 *K. pneumoniae* isolates) as compared to other regions. There is a high probability that specific data by a single center can lead to a resistance rate increase that is not representative for the entire continent. Another reason was that we could not acquire correct information is the rarity of reports from Africa. To enhance the surveillance of bacterial resistance in developing regions is imperative for future infectious disease management. Non-susceptibility of meropenem against *K. pneumoniae* was 8.4 and 10.5% in Latin America and Asia, respectively, in our study, which is similar to the results of SENTRY [11, 12]. However, there is a large difference between our data for North America(2.0%) and the SENTRY data for this region (10.8%) [13].

Similar results were obtained on the susceptibility of cephalosporins and pip/taz to *E. cloacae* comparing our results to the SENTRY report [12]; 61.6–78.4% of cephalosporin-susceptible *E. cloacae* indicated the prudent use of cephalosporins in *E. cloacae* bacteremia. The results of our antimicrobial susceptible tests should be used as a reference. For *S. marcescens* bacteremia*,* there are several antibiotics available for empirical antibiotic treatment.

High in vitro activity was observed for minocycline against Enterobacteriaceae, but it was lower against ESBL-producing *K. pneumoniae.* Poor levofloxacin susceptibility exists in *E. coli* and *K. pneumoniae* (65.2 and 77.5%, respectively), which is worse in ESBL-producing *E. coli* and *K. pneumoniae*(16.3 and 40.5%, respectively),but better in *E. cloacae* and *S. marcescens*(88.1 and 95.2%, respectively). Thus, levofloxacin could be used to treat bacteremia caused by *E. cloacae* or *S. marcescens,* but not in bacteremia caused by *E. coli* or *K. pneumoniae.*

Susceptibility to carbapenem in *A.baumannii* has also been closely monitored. Consistency has been demonstrated by a 31.2% of susceptibility in this investigation and less than 30% from SENTRY [12] and SMART [17] in Latin America. The clinical effect of carbapenem against *A. baumannii* is very limited because the epidemic caused by the global emergence of multi-drug resistant(MDR) isolates. The separation rate of MDR *A. baumannii* between 2004 and 2014 was calculated by Anna et al. [18]. The result was 44.3% globally and up to 60–70% in Latin America, Middle-East, and Africa. Strong resistance is challenging for clinical disease management. The emergence of MDR *A. baumannii* in bloodstream infections significantly increased the mortality in patients [19]. Only minocycline has a better activity against *A. baumannii* in our study(70.8% susceptible isolates), which was similar to that in the SENTRY report (79.1%) [20]. The meta-analysis by Lashinskyet al. [21] on several clinical investigations found that the treatment success rate of minocycline single and combination on *A. baumannii* was 78.2%,verifying the recommendation to use minocycline treatment for an infection caused by *A. baumannii.*

The susceptibility for six antibiotics by *P. aeruginosa* were 71.1–94.9% globally, and 66.7–91.7% and 54.4–75.4% in Asia and Latin America, respectively, which was higher than TEST result (63.9–90.2%) of 2004–2013 [15] for all specimen globally collected, and the SENTRY result for Asia-Pacific(66.7–91.7%) [11] and Latin America(54.4–75.4%) [22] in 2011. *P. aeruginose* isolated from blood may be more susceptible to common antibiotics than the specimens collected from other body sites.

Best in vitro activity was found for linezolid, vancomycin, tigecycline and minocycline against *S. aureus*. Very poor penicillin-susceptibility was observed for *S. aureus,* only 14.7%, which was in accordance with several other investigations [15, 23]. The rate of 33.0% MRSA isolates in globally of our study agreed with 40.2% in the TEST report for 2004–2013 [15]. The isolate rates of MRSA in Asia-Pacific [11], Latin America [12], and the USA [13] were 37,44.7, and 53.1% in the SENTRY surveillance report. In a comparison with our study, the rate for the USA was higher than that for North America(37.7%), whereas the Asia-Pacific rate was lower than the Asia rate (46.6%). A meta- analysis on *S. aureus* in Africa summarized by Matthew [24] found <50% separation of MRSA, which is a smaller difference to our study. Further, 21.2% isolation of MRSA by the EARS-Net surveillance network in Europe is close to 28.9% in our study.

In our investigation, the susceptibility to primary drugs was significantly lower in *E. faecium* than in *E. faecalis,*except for linezolid, tigecycline and minocycline. High resistance levels are a complication during infection treatment. Resistance to vancomycin was significantly higher in *E. faecium* (27.6%) than in *E. faecalis*(1.4%). In VR E. *faecium,* it increased to 61.6 and 58.1% in North America and Latin America, respectively, which is in accordance with the result for the USA (74.7%)[13] and Latin America(50.3%) [12] in the SENTRY surveillance report.

The global occurrence rate of PRSP was 9.0%, and 25.8,13.0,11.8,7.5, and 9.6% in Asia, Africa, Latin America, Europe, and North America, respectively, which was lower than the TEST data from 2009 to 2012(14.4% globally; and 33.1,32.3,15.1,10.1, and 16.3% in the respective regions) [25]. The difference may be linked to the source specimens used for recovering the isolates. Isolates from blood may be more susceptible than these from other body sites. The same situation may apply to other antibiotics. Susceptilbility to macrolides, clindamycin, meropenem and minocycline was 47.5–47.9, 70.2, 83.4, and 51.7%, respectively, in the TEST study from 2009 to 2012 [25], which was significantly less than these in our study. The resistance is higher against many antibiotics in PRSP as compared to that in *S. pneumoniae*, which has been verified by other investigations [26]. The highest activity was determined for linezolid, vancomycin, tigecycline, and levofloxacin irrespective of the resistance against penicillin.

Many research groups investigated and monitored the emergence of macrolide resistance in *S.agalactiae.* A resistance level of even less than 20% is serious for *S.agalactiae,* which is still susceptible to almost all antibiotics [27, 28]. It is very unfortunate that we did not detect the activity of macrolides in our study. *S. agalactiae* is fully susceptible to penicillin, which is critical for decolonization management in pregnant women and infections in other patients. However, the resistance to penicillin is emerging. In a study on *S.agalactiae* isolates from 2012 by Crespo et al. [28], 2% of penicillin-resistant *S. agalactiae* were detected. The mechanism of penicillin resistance in *S.agalactiae* is related to changes in penicillin-binding proteins(PBPs) [29], based on an investigation of resistant strains isolated from cattle.

Tigecycline has the highest in vitro activity against bacteria recovered from blood. Susceptibility to tigecycline in Gram-positive bacteria, including MRSA, VR E. *faecalis,* and PRSP, was above 99.4, except for VR E. *faecium* (98.9%)*.* In Gram-negative bacteria, the susceptibility was high in *E. coli*, and more than 95% in *S. marcescens* and *E. cloacae,* but lower in *K. pneumoniae*(94.8%) and ESBL-producing *K. pneumoniae*(90.4%). Tigecycline was the only antibiotic with over 90% susceptibility in all major pathogens(except for *P.aeruginosa* because of its natural resistance) isolated from blood. High susceptibility values and the difference in various organisms were similar in the SENTRY report from 2016 [30], indicating the reliability of our results and the stability in the efficacy of tigecycline.

The data in Table 5 show that Africa, Asia and Latin America have a serious problem with antimicrobial resistance. There is a higher probability for the people in these regions that they have acquired an antibiotic-resistant organism if they fall ill with a bloodstream infection. Both difficult clinical management and high mortality increase the hardship. Hence, it is imperative to implement rational usage guidelines for antibacterial agents to reduce the occurrence and control the spread of antimicrobial-resistant bacteria globally and especially in the developing countries.

## Conclusion

In our study, tigecycline was the only antibiotic associated with susceptibility in over 90% of all major pathogen isolates collected from blood specimens. In the comparison that included all world regions, the occurrence of antimicrobial-resistant bacteria was higher in Africa, Asia and Latin America than in Europe and North America. Therefore, reinforcement of the surveillance is important in all regions,but it is very critical in developing countries.

## Abbreviations

CARB-NS: Cabapanem non-susceptibility
CLSI: Clinical and Laboratory Standards Institute
ESBL: Extended-spectrum β-lactamase
FDA: US Food and Drug Administration
ICU: Intensive care unit
IHMA: International Health Management Associates
KPC: *K. pneumonia* carbapenemases
MBL: Metallo-β-lactamases
MDR: Multi-drugs resistance
MIC: Minimum inhibitory concentration
MRSA: Methicillin-resistance *S. aureus*
PBP: Penicillin-binding protein
PRSP: Penicillin-resistance *S.pneumoniae*
TEST: Tigecycline Evaluation and Surveillance Trail
VR: Vancomycin resistant
